# Perceptions of quality across the maternal care continuum in the context of a health financing intervention: Evidence from a mixed methods study in rural Malawi

**DOI:** 10.1186/s12913-017-2329-6

**Published:** 2017-06-08

**Authors:** Christabel Kambala, Julia Lohmann, Jacob Mazalale, Stephan Brenner, Malabika Sarker, Adamson S. Muula, Manuela De Allegri

**Affiliations:** 1 Institute of Public Health, Faculty of Medicine, Heidelberg University, Im Neuenheimer Feld 324, D-69120 Heidelberg, Germany; 2 School of Public Health and Family Medicine, College of Medicine, University of Malawi, Private Bag 360, Chichiri, Blantyre 3, Malawi; 3 Environmental Health Department, The Malawi Polytechnic, University of Malawi, Private Bag 303, Chichiri, Blantyre 3, Malawi; 4 James P. Grant School of Public Health, BRAC University, 66 Mohakhali, Dhaka, 1212 Bangladesh

**Keywords:** Results-Based Financing, Malawi, Quality of care, Demand-side financing, Performance-based financing, Maternal care, Conditional Cash Transfers

## Abstract

**Background:**

In 2013, Malawi with its development partners introduced a Results-Based Financing for Maternal and Newborn Health (RBF4MNH) intervention to improve the quality of maternal and newborn health-care services. Financial incentives are awarded to health facilities conditional on their performance and to women for delivering in the health facility. We assessed the effect of the RBF4MNH on quality of care from women’s perspectives.

**Methods:**

We used a mixed-method prospective sequential controlled pre- and post-test design. We conducted 3060 structured client exit interviews, 36 in-depth interviews and 29 focus group discussions (FGDs) with women and 24 in-depth interviews with health service providers between 2013 and 2015. We used difference-in-differences regression models to measure the effect of the RBF4MNH on experiences and perceived quality of care. We used qualitative data to explore the matter more in depth.

**Results:**

We did not observe a statistically significant effect of the intervention on women’s perceptions of technical care, quality of amenities and interpersonal relations. However, in the qualitative interviews, most women reported improved health service provision as a result of the intervention. RBF4MNH increased the proportion of women reporting to have received medications/treatment during childbirth. Participants in interviews expressed that drugs, equipment and supplies were readily available due to the RBF4MNH. However, women also reported instances of neglect, disrespect and verbal abuse during the process of care. Providers attributed these negative instances to an increased workload resulting from an increased number of women seeking services at RBF4MNH facilities.

**Conclusion:**

Our qualitative findings suggest improvements in the availability of drugs and supplies due to RBF4MNH. Despite the intervention, challenges in the provision of quality care persisted, especially with regard to interpersonal relations. RBF interventions may need to consider including indicators that specifically target the provision of respectful maternity care as a means to foster providers’ positive attitudes towards women in labour. In parallel, governments should consider enhancing staff and infrastructural capacity before implementing RBF.

## Background

Poor quality of maternal healthcare services has been recognised as the major factor contributing to maternal and newborn deaths in most low and middle income countries (LMICs) [1]. Insufficient human resources [2, 3], poor training of health staff [2], inadequate infrastructures, and shortages in equipment and medications have been identified as the main causes of structural inadequacies contributing to poor health service delivery [3, 4]. In turn, it has been argued that these inadequacies are largely the product of both insufficient and inefficient health financing structures [5]. Specifically, it has been postulated that input-based financing coupled with excessive centralization exacerbates the constraints already imposed by scarcity of resources and ends up depriving healthcare workers of the autonomy needed to make decisions that are conducive to the delivery of quality health services in these settings [6].

Performance Based Financing (PBF) has been advanced as a health system intervention able to improve healthcare service delivery in LMICs [7]. Specifically, PBF is expected to produce changes in quantity and quality of service delivery by promoting a shift from input-based to output-based financing [7, 8]. Under PBF, health-care providers, health facilities, and district health management teams enter in a contractual arrangement with a purchasing agent (either the government or a development partner) and are financially rewarded only upon reaching predefined performance targets related to the quantity and quality of the healthcare services provided [7–9]. Under PBF, both quantity and quality outputs are verified on a regular basis [7]. The shift from input-based to output-based financing is further coupled with increased autonomy, allowing healthcare providers to make independent choices about resource allocation at the level of their facilities [10, 11].

Based on principal-agent theory, it is expected that linking payments to specific quantity and quality outputs can more easily re-align healthcare workers’ behaviour towards the provision of quality services than input-based financing ever did [13, 14]. In addition, increased autonomy is expected to empower healthcare providers to overcome health system barriers that are not conducive to quality health service delivery by allowing them to make independent decisions about the investments that can best benefit the facility they work at and the community they serve [10, 11]. Last, but surely not least, verification is expected to make healthcare providers more accountable both towards the communities and the government they work for, in countries where healthcare employees have traditionally lacked accountability [7, 12].

Over the last decade, several LMIC governments have adopted PBF, either alone or in combination with demand-side financing interventions, such as Conditional Cash Transfers (CCT) [7, 10, 13–18]. CCT are financial incentives acting on the demand-side to motivate consumption of specific healthcare services. While PBF acts on the supply-side by rewarding provision of a given service at a set quality standard, CCT act on the demand-side by rewarding consumption of a given service [7, 9]. When used in combination, PBF and CCT are expected to produce changes on both supply and demand, resulting in increases both in the quantity and the quality of the services delivered [7]. There have been concerns raised against the provision of CCT. If demand for a given service be increased beyond the current health system capacity, it will potentially counteract the positive effect on quality of service delivery promoted by PBF [19].

With specific reference to maternal care services, which represent the focus of our investigation in this paper, PBF has been shown to improve use and quality of maternal and child health services in Burundi [20]; and to improve utilization, coverage and emergency referrals with enhanced quality of provider performance in Haiti [21]. PBF has also been shown to increase rates of assisted births, antenatal care utilization and uptake of modern family planning in Burundi [22]; to increase utilization and coverage of maternal services in India, Kenya and Uganda [14]; and to increase the number of institutional deliveries in Rwanda [13]. Further, PBF has been shown to improve structural quality such as an increase in the availability of staff in Democratic Republic of Congo [10]; and to improve process quality such as history taking, examination of pregnant women, testing blood and urine during ANC in Egypt [23] to name a few.

Additional evidence, however, has indicated that the provision of financial incentives depending on performance is not always sufficient to improve the quality of the care provided [6, 24] and may result in negative outcomes on structural quality, such as a decrease in the level of availability of equipment and drugs [25], may lead to neglect of untargeted services [6, 24], provision of needless or detrimental services and fraud [24, 26]. On the demand side, CCT linked to provision of maternity services are treated with caution, albeit the absence of concrete evidence, due to the fear that they may encourage women to have additional children and to decrease access to unrewarded interventions [6].

The review presented above clearly indicates that knowledge on the effects of PBF on quality of service delivery is still limited. In addition, the studies reviewed focused exclusively on a quantitative assessment of structural and process dimensions [25] measured at the health facility level. Little attention has so far been paid to understanding if and how the experiential dimension of quality, that is to say the quality of service delivery as experienced directly by consumers, changes as a function of the introduction of PBF in a given setting. Since quality of care is widely recognized to be a multidimensional [1, 27] construct, the exploration of experiential dimensions deserves the same attention as the exploration of structural and process elements [28]. Specifically, according to Wilde et al. (1993), “clients’ perceptions of good quality care are formed by the intersection between resources available to the health service organisation and the patient preferences considered from four dimensions: the medical-technical competence of the caregivers, the physical-technical conditions of the care organization, the identity-orientation in the attitudes and actions of the caregivers, and the socio-cultural atmosphere of the care “[29]. Thus, a full understanding of the effect of PBF on quality of care cannot be limited to quantitative assessment of structural and process elements, but needs to account for an analysis of these experiential elements as well. Further, little is known on the effect of incentives in relation to women’s experiences and perceptions of quality of care on technical care, quality of amenities and interpersonal relations.

Our mixed-methods study aimed to fill this gap in knowledge, by assessing the effect of an intervention combining PBF and CCT on women’s experiences and perceptions of the quality of maternal and neonatal care services delivered in rural Malawi.

### Study setting

This study was conducted in Malawi where a Result Based Financing Initiative (combining PBF and CCT) (described below) is being implemented as a strategy to improve the utilization and the quality of maternal and newborn healthcare services. Malawi is a low income country in sub-Saharan Africa (SSA) and is located to the south of the equator [30]. The country is divided into three regions: the Northern, Central and Southern regions. Each region is divided into districts and there are 28 in total [31]. Estimates in 2015 indicated that the population of Malawi was at 17,261,736 million [32].

Malawi is among the countries with highest maternal and neonatal mortalities worldwide with a Maternal Mortality Ratio (MMR) of 574/100, 000 live births and a Neonatal Mortality Rate (NMR) of 29/1000 live births as estimated in 2015 [31]. A weak health system and poor quality of care are recognized as the main factors contributing to continuing high rates of maternal and neonatal deaths [33]. Poor quality of care is mostly attributed to an inequitable distribution of health facilities, poor functioning of the referral system, poor structural amenities, often non-functional equipment, poor or no supply of drugs and other essential supplies, as well as few skilled birth attendants [33–35]. Compounding the problem are factors that hinder access to maternal health services, such as long distances to health facilities and lack of finances for transportation [35–38]. Other hindrances include lack of finances for purchasing delivery related items (i.e. women may fear to go to the health facility without satisfying the requirements for an expectant mother to bring delivery related items e.g. wrappers for the baby, a basin for bathing the baby and a plastic sheet to be used in place of a Mackintosh roll [waterproof sheet] on the delivery bed) and upkeep while in the facility [35–38]. Furthermore, the literature consistently indicates poor provider-patient interactions, whereby women report rude and disrespectful treatment during child-birth [36, 37]. In spite of all these hindrances, 96% of women receive antenatal care (ANC) at a health facility at least once, 45% use ANC services at least four times, 90% deliver in a health facility and 81% receive postnatal care (PNC) within 2 days of delivery [31].

### Results Based Financing for Maternal and Newborn Health (RBF4MNH) Initiative in Malawi

In 2013, the Ministry of Health (MoH) of Malawi adopted the Results-Based Financing for Maternal and Newborn Health (RBF4MNH) Initiative. The aim of the Initiative is to improve the quality of maternal and newborn care services while maintaining high service utilization in both public and selected private not-for-profit facilities. The MoH implements the Initiative through its Reproductive Health Unit (RHU), with funding from the Norwegian and German governments and with technical support from Options Consultancy Services Limited [39, 40]. The RBF4MNH Initiative is being implemented in 4 (i.e. Balaka, Dedza, Ntcheu and Mchinji) out of 28 districts in Malawi. The four districts together account for about 13.26% (2000000) of the population of Malawi [31]. Before the launch of the Initiative, the MoH conducted a feasibility assessment and identified all facilities able to perform all required emergency obstetric and neonatal care (EmONC) signal functions (essential medical interventions for handling complications in the labour ward). Based on this assessment and on geographical location, in 2013, the MoH selected 18 facilities, where to roll out the intervention and 1 year later (i.e. in 2014) added 10 more facilities. Alongside Basic EmOC facilities, all four public (one per district) and one private not for profit Comprehensive EmOC facilities have been included in the intervention already since 2013 [40].

The RBF4MNH Initiative includes both supply (provision of obstetric services) and demand side (utilization of obstetric services) incentives. The supply side intervention (PBF) comprises financial rewards that are provided to health facilities upon attainment of a predefined set of indicators pertaining to clinical and organizational performance during labour, delivery, and newborn care [39]. For example, besides many indicators on clinical performance (e.g. use of partographs during childbirth), quality assurance (e.g. record audits) and service management (e.g. equipment maintenance), RBF4MNH facilities conduct patient satisfaction surveys (exit interviews) on a monthly basis with at least 10 women. Independent staff (other than those providing care) in the facilities asks women about their satisfaction with services and provider attitudes. Then, providers choose one issue highlighted by patients in the survey and devise an action plan to mitigate the raised concern. During verification of other indicators on clinical and organizational performance, verifiers also check if health facilities have conducted surveys on client satisfaction. If facilities do not have documentation to prove that they have spoken (surveyed) to at least 10 women, this is documented during verification activities and facility rewards are negatively affected. Of the total financial rewards received on a quarterly basis, 30% are to be invested into the health facility; the remaining 70% can be paid out to staff as bonuses. At intervention facilities, initial investments were made into facilities’ infrastructure and equipment to ensure minimum standards for provision of quality maternal and neonatal health-care services before the intervention was rolled out [39]. Further to this, healthcare providers in intervention facilities were trained (refresher courses) in antenatal management, obstetric care and quality assurance. In addition to the incentives being directed towards the single facilities, District Health Management Teams are also rewarded based on the overall performance of a district, including both RBF4MNH and non-RBF4MNH facilities. This set of incentives is meant to ensure that that management teams continue to perform adequate supervision across all facilities and do not re-direct resources exclusively towards intervention facilities.

The demand side intervention comprises conditional cash transfers (CCT) to women who deliver in a health facility. A fixed lump sum is provided to recover upfront costs of delivery-related items (e.g. wrappers for the baby) and food while staying at the facility for 48 h after delivery. In addition, a variable sum is reimbursed for transport depending on distance travelled [39]. Receipt of the CCT is conditional upon having already registered at the facility during antenatal care, with Health Surveillance Assistants verifying women’s eligibility by checking on their actual village of residence.

In line with the overall theory of change of PBF described in the introduction, the RBF4MNH Initiative aims at increasing utilization of obstetric services (facility-based delivery and 48-h stay post-partum) through the application of CCT and quality of service delivery through the application of PBF with incentives being closely tied to specific quality targets (the list is attached as an appendix).

## Methods

### Study design

Our study was conducted within the framework of a larger evaluation set to assess the impact of the RBF4MNH Initiative (including all of its elements, PBF and CCT) on a wide range of indicators related to utilization and quality of maternal and newborn care [39]. The evaluation relied on a mixed-methods prospective sequential controlled pre- and post-test design with independent controls, whereby we integrated quantitative and qualitative methods of data collection and analysis within a single design [39]. The choice of study design was determined in light of the fact that the intervention facilities were purposely (and not randomly) selected by the MoH among the complete set of EmOC facilities in the four concerned districts. As controls, we used the EmOC facilities sampled during the MoH baseline feasibility assessment, but later not included in the intervention. This ensured comparability between intervention and control facilities, since the two shared a set of basic characteristics linked to their capacity to perform EmOC functions. The 2014 expansion of the RBF4MNH Initiative described earlier, however, meant that our count across intervention and control facilities changed over time. At the onset of the project, we had 18 intervention and 15 control facilities, while at end-term we counted 23 intervention and 10 control facilities. Thus, 1 year into our study (after midterm data collection), five facilities initially included as controls switched to the intervention group due to a natural scale up of the intervention.

We collected data prospectively following the intervention rollout over a 3-year period: at baseline (before the intervention began), at mid-term (a year into the implementation of the intervention), and at end-term (2 years into the implementation of the intervention). We collected quantitative data at all three time-points, while, in line with our sequential design [41], we collected qualitative data only at mid-term and end-term. Although the intervention specifically targeted labour and delivery services, our evaluation efforts addressed the complete range of services along the maternal care continuum. Our approach was justified by a wish to account for both the expected and unexpected effects of the Initiative on pregnant and labouring women and their babies.

With specific reference to the study component described in this manuscript, quantitative data were used to quantify changes in women’s experiences and perceptions over time, while qualitative data were used to explore experiences and perceptions in greater depth, providing a better understanding and contextualization of the emerging quantitative findings [42]. Both our quantitative and qualitative tools were used to measure perceived quality of care in relation to three dimensions (technical care, quality of amenities and interpersonal relations) identified as essential components of perceived quality of care (QoC) in the literature [29], as already highlighted in the introduction.

### Quantitative instrument, data collection strategy, and sample

In line with the overall design of the impact evaluation [39], we collected quantitative data at three time-points by means of a repeated cross-sectional survey conducted in 2013 (baseline), 2014 (mid-term), and 2015 (end-term) among women exiting maternal and neonatal care services at all 33 health facilities included in the study.

Due to feasibility concerns, we relied on convenience sampling techniques to recruit women for the exit interviews. Each year, over the designated data collection period, we stationed at each facility included in the study for 3 to 5 days and during these days, we approached women exiting ANC, labour and delivery (L&D), and PNC on a continuous basis. We explained the aim of our study and interviewed all women who agreed to take part in it. To ensure sufficient analytical power, we aimed at interviewing at least eight women for each set of services (ANC, L&D, and PNC) at each concerned facility and in each survey round (i.e. baseline, mid-term and end-line). This yielded a total sample of 3068 (1407 for ANC; 766 for L&D; and 895 for PNC). Of the total sample, 2041 women were interviewed at intervention and 1027 at control facilities with an approximately equal number of interviews per data collection round.

We used structured close-ended questionnaires that only differed in some details depending on the service cohort surveyed (i.e. ANC, L&D, PNC) to collect information on demographic and socio-economic characteristics of the participants, women’s experiences with receiving maternal care at the facility (i.e. recall of which services they received during their visit to the facility), and their perceptions of the quality of care they received. Interviews were administered face-to-face by trained enumerators using digital data collection devices under the direct supervision of the authors.

### Quantitative outcome variables

We estimated the impact of RBF4MNH on two sets of outcome variables: women’s experiences of maternal care services received during their facility visits; and women’s perceptions of the quality of these services.

The first set of outcomes variables, women’s experience with maternal care, pertains to women’s recall of the exact services they received during the provider-patient encounter that had concluded just prior to our interview. We measured women’s experience with care by asking whether they had or had not a number of routine elements in the service delivery process central to technical and interpersonal service quality of ANC, L&D, PNC: health worker introduction, having been examined, getting an explanation for the examination, having diagnostic tests, getting an explanation for the diagnostic tests, having a blood pressure check, requested consent before procedures, having received medications/treatment, getting an explanation for medication purpose, encouraged to ask questions, offered to have a guardian during delivery and, privacy/confidentiality protection. Variables were coded as (1) if the woman indicated to have received or experienced a certain treatment and (0) if not (Table 1).

**Table 1:** Variables on women’s experiences with receiving care and their measurement

Indicator	Measurement
Health worker introduction	0 = Not done 1 = Done
Examinations/clinical procedures conducted	0 = Not done 1 = Done
Explanation of examination/clinical procedures	0 = Not explained 1 = Explained
Medications administered	0 = Not administered 1 = Administered
Explanation of dosage and purpose of medication	0 = Not explained 1 = Explained
Blood specimen collected	0 = Not collected 1 = Collected
Explanation of the purpose of the blood specimen collected	0 = Not explained 1 = Explained
Consent sought before procedures	0 = Not sought 1 = Not sought
Encouraged to ask questions	0 = Not encouraged 1 = Encouraged
Encouraged to have a guardian	0 = Not encouraged 1 = Encouraged
Privacy and confidentiality protected	0 = Not protected 1 = Protected
Blood pressure taken during ANC or PNC	0 = Not taken 1 = Taken
Blood pressure taken before delivery	0 = Not taken 1 = Taken
Blood pressure taken after delivery	0 = Not taken 1 = Taken
Baby weight checked during PNC	0 = Not checked 1 = Checked

The second set of outcome variables pertains to women’s perceptions of the quality of the services they received during the provider-patient encounter that had concluded just prior to our interview. In line with our conceptual model which reflects the theoretical postulations on quality outlined by Wilde et al. (1993) [29] to which we refer earlier in the manuscript, we looked at perceived quality on three dimensions for all three services: technical care (i.e. technical aspects of maternal care), quality of amenities, and interpersonal relations. Quality perceptions included the above service elements for which we inquired women’s experiences, but went beyond. Perceived quality was measured with psychometric 10-point Likert scales that encompassed a series of brief statements. We developed three parallel, yet distinct surveys (and scales), each addressing the abovementioned dimensions of quality of care with reference to the specific services delivered during ANC, L&D, and PNC services. The scales for ANC and PNC each encompassed 27 parallel statements, for example, ‘The health worker listened to me’, ‘She/he behaved in a gentle manner’, ‘The room was clean and hygienic’. For L&D, we added four statements to those used for ANC and PNC to capture additional service aspects, for example, “The health worker explained the process of labour and delivery”, “The health worker was attentive towards my baby”. For each respondent, a score was then calculated as the un-weighted mean of a woman’s responses to the statements pertaining to the three quality dimensions, respectively [43]. A detailed description of the measurement and calculation of the QoC scores is provided elsewhere [43]. In the analyses, perceived QoC scores are treated as continuous variables. The full list of statements is provided in Table 2 and questionnaires are attached.

**Table 2:** Variables used for composite scores for each perception aspect of care adapted to each service cohort

Quality of care dimensions measured on a scale of 1–10 (1 = complete disagreement; 10 = complete agreement)	
Perception of technical care aspects of received care	I felt confident in the health worker’s ability to assist me
	The health workers were competent
	She/he was available for me
	The midwife/birth attendant or health worker was attentive towards my baby
	She/he was supportive with regard to breastfeeding
	She/he looked after my pain(s)
	She/he made sure me and my baby are well
	She/he was with me during labor and delivery
	The health workers were well coordinated
	The health workers were efficient
Perceptions of quality of amenities	Finding the way to the ANC/del/PNC examination room was easy
	The ANC/del/PNC examination room was well equipped
	I was set up comfortably in the ANC/del/PNC examination room
	The ANC/del/PNC examination room was clean and of satisfactory hygiene
	The ANC/del/PNC room was big enough
	The ANC/del/PNC examination room was calm, without noise
	The ANC/del/PNC examination room was not too dark
	The temperature in the delivery room was satisfactory
Perception of interpersonal aspects of received care	The health worker made a good impression on me
	She/he listened to me
	She/he was attentive towards my needs
	She/he behaved in a gentle manner
	She/he spoke in a gentle manner
	Overall, the health workers had respect
	Overall, the health workers were sensitive
	Overall, the health workers were nice to me
	Overall, the health workers were patient
	I believe that people working in this health facility are honest
	She/he explained the process of labor and delivery
	She/he reassured me concerning my worries
	She/he talked in a way that helped me understand my condition

### Statistical analysis

We estimated the effect of PBF on perceived QoC scores and women’s experiences using a difference-in-differences (DID) regression model. We relied on two different models: the first model estimated changes from baseline (2013) to mid-term (2014), while the second model estimated changes from baseline to end-term (2015). The purpose of doing so was to differentiate the short-term effect of the intervention (1 year into its implementation) from its longer-term effect (2 years into its implementation).

As the DID model used in our analysis is based on linear regression, corresponding standard errors were estimated by Ordinary Least Squares (OLS):

$$ {\mathrm{Y}}_{\mathrm{i}\mathrm{t}} = {\upbeta}_0+{\upbeta}_1{\mathrm{T}}_{\mathrm{i}\mathrm{t}}+{\upbeta}_2{\mathrm{A}}_{\mathrm{i}}+{\upbeta}_3{\mathrm{T}}_{\mathrm{i}}{\mathrm{A}}_{\mathrm{i}}+{\upvarepsilon}_{\mathrm{i}\mathrm{t}}, $$

Where Y_it_ = outcome variable (i.e. QoC and women’s experience); T_it_ = 1 if observation i occurred in treated facilities and 0 otherwise; A_i_ = 1 for follow-up time points (mid-term, end-term) after treatment occurred and 0 otherwise and; T_i_A_i =_ interaction between treatment and time points.

We adjusted both DID regression models to control for women’s age, literacy, socio-economic status (SES; asset index), number of previous pregnancies (gravidity), and a variable that identified the facilities which transitioned from comparison to intervention shortly following our mid-term data collection. The selection of these control variables was informed by previous analyses exploring heterogeneity in our outcome variables [43], to include variables that could potentially mediate the effect of the intervention. Since we observed outcomes at individual level while the intervention was implemented at group level (i.e. health facility), we controlled for clustering at health facility level using the cluster option in Stata [44]. Further, to counteract possible weaknesses in the estimation models due to the limited number of clusters (33), we applied a bootstrapping technique [44–47]. We analysed the data using Stata IC version 13 (StataCorp LP, Texas)**.**

### Qualitative procedures

In line with the overall study design, we collected qualitative data in 2014 (mid-term) and 2015 (end-term). Our qualitative data collection procedures relied on an emergent design, with somewhat different decisions taken at mid-term and at end-term, in light of the emerging quantitative findings. At mid-term, we purposely targeted both intervention (*n* = 8) and control (*n* = 4) facilities, while at end-term, we exclusively targeted intervention facilities (*n* = 12). This difference in sampling choices is due to the fact that while at mid-term, we wished to explore differences between control and intervention areas in relation to the experiential dimension of care, at end-term, we were more interested in exploring the heterogeneity of experiences in relation to the implementation of the RBF4MNH Initiative. Both at mid-term and at end-term, we selected facilities based on how the women included in our quantitative sample had judged their performance, sampling facilities whose quality of service delivery had been rated as either high (≥6/10) or low (<6/10). The application of this purposeful criterion was made possible by the sequential nature of our design, with qualitative data collection being informed by emerging quantitative findings.

**Fig. 1:**
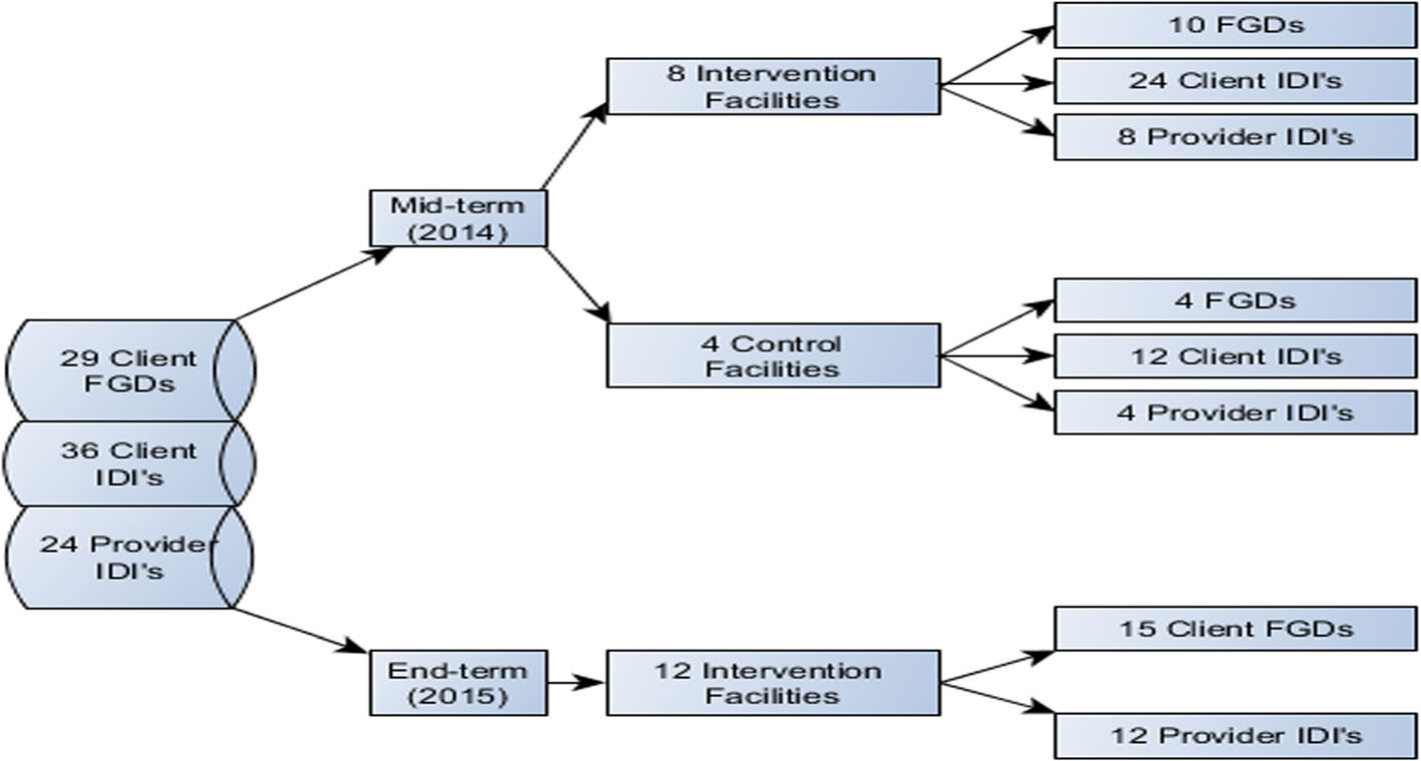
Qualitative Sampling Design

At mid-term, we conducted both in-depth exit interviews (IDI) with women exiting ANC, L&D and PNC services and Focus Group Discussions (FGD) with larger groups of women in communities directly served by the selected facilities. Women selected for FGD included only those who had accessed maternal care services at the selected facilities during the RBF4MNH implementation period. Since during the mid-term data collection round, we observed greater willingness to talk about experiences of care in a collective (i.e. FGD) rather than in an individual setting (i.e. IDI), at end-term we maintained only the FGD as means of data collection. In addition, for triangulation purposes, we interviewed one maternity health-care provider at each of the selected facilities (Fig. 1 indicates total samples). Data collection was continued until saturation and redundancy were reached. While the number of facilities was sampled in advance, we did not determine the number of interviews and FGD in advance to ensure that we could reach saturation.

The interview guides for both the IDI and the FGD were largely developed to reflect the three dimensions of care (technical care, quality of amenities and interpersonal relations) measured also by our quantitative tool. Trained qualitative interviewers conducted the interviews and led the FGDs in Chichewa (the local language) under the supervision of the corresponding author. All interviews and discussions were audio-recorded, transcribed verbatim, and translated into English before analysis.

### Qualitative data analysis

We analysed data using content analysis which relied on a directed approach to coding [48]. First, we developed an initial set of codes based on the conceptual understanding of quality described earlier and on specific elements emerging from a preliminary analysis of the quantitative findings. Second, we let additional codes emerge as we identified new relevant themes while we proceeded through the reading. Third, we grouped similarly coded portions of text according to emerging over-arching themes. Last, we looked for connections and inter-relations across themes to eventually construct a comprehensive narrative [49]. Three analysts coded the material independently and later discussed findings to reach a common interpretation of the material (analysts’ triangulation). Any discrepancy identified was resolved by returning to the transcribed material for additional analysis. Analysis was carried out with support of QSR NVivo [50].

The final interpretation of the data emerged in discussion with the broader research team to appraise findings in the light of the results of the wider impact evaluation.

## Results

### Quantitative findings

#### Characteristics of women attending L&D, ANC and PNC care

Table 3 reports results, by differentiating them across the three sets of respondents (ANC, L&D, and PNC) and across intervention and control facilities. In the absence of randomization, this comparison was needed to ensure comparability between women interviewed at intervention and at control facilities, before we proceeded with the DID regression model. Across intervention and controls, women’s age in the L&D sample was 24.0 years (SD: 5.6), in the ANC sample was 24.7 years (SD: 5.9) and in the PNC sample was 24.2 years (SD: 5.6). Overall, close to 96% of women were married and close to 65% of women reported being literate. We observed no significant differences in women’s gravidity between the intervention and controls for the L&D sample and for the ANC sample, with most women reporting having had two pregnancies. Only among PNC respondents, women at comparison facilities reported a higher number of prior pregnancies (3.1, SD: 2.0) than women at intervention facilities (2.6, SD: 2.0).

**Table 3:** Sample characteristics of women exiting L&D, ANC and PNC services

Characteristic	Baseline				Midterm				Endline				*T*-test	
	Comparison		Intervention		Comparison		Intervention		Comparison		Intervention			
	*n* = 067		*n* = 136		*n* = 109		*n* = 224		*n* = 040		*n* = 190		*t*	Sig
Women exiting L&D services														
Average age in years (mean/SD)	25.1	6.0	24.3	6.0	24.1	4.7	24.0	5.5	22.6	4.3	23.9	5.9	0.3	0.79
Proportion of married women (*N*/%)	61	91.0	128	94.1	103	94.5	218	97.3	39	97.5	188	99.0	−2.0	0.04*
Proportion of literate women (*N*/%)	51	76.1	83	61.0	73	67.0	140	62.5	24	60.0	123	64.7	1.5	0.14
Average number of pregnancies (mean/SD)	3.1	1.7	2.6	1.8	2.2	1.8	2.5	2.0	1.8	2.0	2.2	2.1	0.0	0.97
Average number of living children (mean/SD)	2.8	1.6	2.4	1.6	2.1	1.7	2.3	1.9	3.1	0.9	3.2	1.2	0.1	0.95
Proportion with previous miscarriage (*N*/%)	9	13.4	14	10.3	11	10.1	20	8.9	2	5.0	18	9.5	0.3	0.76
Proportion with previous stillbirth (*N*/%)	1	1.5	5	3.7	2	1.8	10	4.5	1	2.5	6	3.2	−1.4	0.17
Proportion with previous premature birth (*N*/%)	5	7.5	7	5.2	2	1.8	13	5.8	2	5.0	10	5.3	−0.7	0.47
Women exiting ANC services	*n* = 167		*n* = 221		*n* = 250		*n* = 365		*n* = 99		*n* = 305			
Average age in years (mean/SD)	25.3	6.5	24.2	5.8	24.2	5.8	25.0	5.7	24.5	5.4	25.0	6.0	−0.6	0.54
Proportion of married women (*N*/%)	163	97.6	216	97.7	249	99.6	356	97.5	96	97.0	289	94.8	0.8	0.44
Proportion of literate women (*N*/%)	111	66.5	146	66.1	166	66.4	243	66.6	52	52.5	193	63.3	−0.6	0.56
Average number of pregnancies (mean/SD)	3.2	2.1	2.8	1.8	1.9	1.8	2.1	1.8	3.0	1.7	2.9	1.8	−0.3	0.79
Average number of living children (mean/SD)	1.9	1.9	1.5	1.6	1.7	1.6	1.8	1.6	1.8	1.5	1.7	1.6	0.7	0.46
Proportion with previous miscarriage (*N*/%)	23	13.8	32	14.5	23	9.2	50	13.7	12	12.0	30	9.8	−0.7	0.46
Proportion with previous stillbirth (*N*/%)	9	5.4	8	3.6	11	4.4	14	3.8	1	1.0	13	4.3	0.1	0.90
Proportion with previous premature birth (*N*/%)	9	5.4	14	6.3	10	4.0	11	3.0	3	1.9	10	3.3	0.2	0.82
Women exiting PNC services	*n* = 080		*n* = 150		*n* = 138		*n* = 220		*n* = 077		*n* = 230			
Average age in years (mean/SD)	25.5	6.4	24.7	5.6	23.3	5.0	24.5	6.0	24.8	6.1	23.7	5.6	0.1	0.94
Proportion of married women (*N*/%)	78	97.5	139	92.7	135	97.8	212	96.4	73	94.8	220	95.7	1.2	0.21
Proportion of literate women (*N*/%)	50	62.5	91	60.7	90	65.2	150	68.2	43	55.8	148	64.4	−0.8	0.41
Average number of pregnancies (mean/SD)	3.1	2.1	2.7	1.9	3.5	1.7	3.6	1.9	3.7	1.8	3.2	1.4	2.9	0.01*
Average number of living children (mean/SD)	2.7	1.7	2.4	1.5	2.4	1.6	2.6	1.8	3.6	1.7	3.1	1.3	1.9	0.05*
Proportion with previous miscarriage (*N*/%)	13	16.3	19	12.7	9	6.5	14	6.4	3	3.9	14	6.1	0.5	0.60
Proportion with previous stillbirth (*N*/%)	5	6.3	4	2.7	6	4.4	4	1.8	2	2.6	7	3.0	1.7	0.09
Proportion with previous premature birth (*N*/%)	4	5.0	11	7.3	6	4.4	7	3.2	6	7.8	10	4.4	0.6	0.52

##### Effect of RBF4MNH on women’s experiences with receiving care during labour and delivery

As displayed in Table 4, we observed no statistically significant change attributable to the RBF4MNH on the vast majority of experience indicators reported by women, the exception being a positive effect at end-term on the probability of receiving medication/treatment (*p* = 0.03) and a negative effect at mid-term on the probability of undergoing a blood test (*p* = 0.03).

**Table 4:** Impact of RBF4MNH on women’s experience of receiving care during labour and delivery

Indicator	Baseline				Mid-term				End-term				DID adjusted BL-ML	Sig.	DID adjusted BL-EL	Sig.
	Comparison		Intervention		Comparison		Intervention		Comparison		Intervention					
	%	*N*	%	*N*	%	*N*	%	*N*	%	*N*	%	*N*				
Proportion of women reporting health worker introduction	26%	53	37%	68	30%	89	38%	120	40%	62	49%	85	−4%	0.77	−4%	0.82
Proportion of women reporting having been clinically examined	57%	53	46%	68	99%	89	96%	120	92%	62	91%	85	5%	0.73	6%	0.75
Proportion of women reporting having received an explanation of the examination	100%	30	100%	31	64%	88	58%	115	54%	57	58%	77	−5%	0.69	−2%	0.85
Proportion of women reporting having received medication/treatment	92%	53	71%	68	87%	89	88%	120	66%	62	79%	85	23%	0.07	29%	0.03*
Proportion of women reporting having received an explanation for the medication	59%	49	63%	48	42%	77	46%	106	56%	41	73%	67	−3%	0.85	12%	0.54
Proportion of women reporting having had a blood test done	26%	53	47%	68	21%	89	8%	120	23%	62	36%	85	−35%	0.03	−2%	0.90
Proportion of women reporting having received an explanation for the test	93%	14	75%	32	32%	19	30%	10	64%	14	65%	31	9%	0.69	10%	0.65
Proportion of women reporting consent being sought before procedures were performed	55%	53	61%	66	62%	87	63%	112	68%	60	75%	77	−5%	0.73	1%	0.96
Proportion of women having been encouraged to ask questions	36%	53	49%	68	25%	89	33%	120	45%	62	58%	85	−8%	0.63	2%	0.89
Proportion of women reporting being offered to have guardian during delivery	42%	53	57%	68	22%	89	58%	120	50%	62	55%	85	18%	0.29	3%	0.87
Proportion of women reporting their privacy/confidentiality being protected	96%	53	96%	68	91%	89	94%	120	90%	62	96%	85	3%	0.67	8%	0.55
Proportion of women reporting blood pressure was checked before delivery	53%	53	51%	68	49%	89	37%	120	61%	62	65%	85	−15%	0.22	19%	0.26
Proportion of women reporting blood pressure was checked after delivery	40%	53	44%	68	66%	89	57%	116	45%	58	68%	81	−13%	0.36	19%	0.25

DID = effect estimate based on difference-and-difference regression; BL-ML = comparison between cohorts at baseline and mid-term; BL-EL = comparison between cohorts at baseline and end-term; Sig. = significance level of effect estimate

##### Effect of RBF4MNH on women’s experiences with receiving care during ANC

As shown in Table 5, we observed no statistically significant change attributable to the RBF4MNH on many of the experience indicators, except for a positive effect at mid-term on the probability of being offered to keep a guardian during ANC consultation (*p* = 0.04) and a negative effect on the probability of receiving a blood pressure check (*p* = 0.01).

**Table 5:** Impact of RBF4MNH on women’s experience of receiving care during ANC

Indicator	Baseline				Mid-term				End-term				DID adjusted BL-ML	Sig.	DID adjusted BL-EL	Sig.
	Comparison		Intervention		Comparison		Intervention		Comparison		Intervention					
	%	*N*	%	*N*	%	*N*	%	*N*	%	*N*	%	*N*				
Proportion of women reporting health worker introduction	34%	152	51%	128	49%	223	53%	244	43%	133	61%	135	−12%	0.37	−9%	0.56
Proportion of women reporting having been clinically examined	98%	152	98%	128	99%	223	96%	244	96%	133	98%	135	−3%	0.19	5%	0.39
Proportion of women reporting having received an explanation of the examination	72%	149	84%	126	67%	221	70%	235	80%	128	83%	132	−7%	0.53	1%	0.94
Proportion of women reporting having received medication/treatment	96%	152	93%	128	86%	223	87%	244	93%	133	93%	135	4%	0.62	−4%	0.43
Proportion of women reporting having received an explanation for the medication	84%	146	86%	119	75%	191	71%	213	90%	124	94%	126	−8%	0.43	2%	0.71
Proportion of women reporting having had a blood test done	74%	152	68%	128	37%	223	36%	244	69%	133	68%	135	6%	0.69	−2%	0.91
Proportion of women reporting having received an explanation for the test	95%	113	94%	87	78%	82	86%	87	96%	92	90%	92	12%	0.18	−1%	0.84
Proportion of women reporting having had any clinical procedures performed	98%	152	100%	128	99%	223	98%	244	100%	133	100%	135	−3%	0.09	−3%	0.11
Proportion of women reporting consent being sought before procedures were performed	68%	149	77%	128	73%	221	74%	239	82%	133	91%	135	−8%	0.41	3%	0.72
Proportion of women having been encouraged to ask questions	61%	152	71%	128	55%	223	58%	244	73%	133	80%	135	−8%	0.52	6%	0.57
Proportion of women having been offered to have a guardian	53%	152	61%	128	36%	223	71%	244	80%	133	70%	135	29%	0.04	−7%	0.60
Proportion of women reporting their privacy & confidentiality being protected	97%	152	99%	128	95%	223	98%	244	94%	133	94%	135	1%	0.65	3%	0.72
Proportion of women reporting blood pressure having been checked	54%	152	82%	128	73%	223	56%	240	69%	130	71%	135	−47%	0.01	−28%	0.10

DID = effect estimate based on difference-and-difference regression; BL-ML = comparison between cohorts at baseline and mid-term; BL-EL = comparison between cohorts at baseline and end-term; Sig. = significance level of effect estimate

##### Effect of RBF4MNH on women’s experiences with receiving care during PNC

We observed no statistically significant change attributable to the RBF4MNH on indicators related to clients’ experience with PNC (Table 6).

**Table 6:** Impact of RBF4MNH on women’s experience of receiving care during PNC

Indicator	Baseline				Mid-term				End-term				DID adjusted BL-ML	Sig.	DID adjusted BL-EL	Sig.
	Comparison		Intervention		Comparison		Intervention		Comparison		Intervention					
	%	*N*	%	*N*	%	*N*	%	*N*	%	*N*	%	*N*				
Proportion of women reporting health worker introduction	40%	63	49%	106	36%	136	34%	142	34%	109	50%	103	−11%	0.59	2%	0.92
Proportion of women reporting having been clinically examined	90%	63	67%	106	74%	136	56%	142	65%	109	74%	103	5%	0.76	35%	0.09
Proportion of women reporting having received an explanation of the examination	68%	57	67%	71	65%	100	60%	80	84%	71	63%	76	−4%	0.80	−20%	0.11
Proportion of women reporting having received medication/treatment	49%	63	42%	106	30%	136	23%	142	44%	109	36%	103	0.2%	0.99	−15%	0.48
Proportion of women reporting having received an explanation for the medication	68%	31	87%	45	63%	41	63%	32	74%	48	64%	37	−20%	0.30	−29%	0.10
Proportion of women reporting having had a blood test done	25%	63	20%	106	4%	136	3%	142	11%	109	20%	103	5%	0.71	4%	0.78
Proportion of women reporting having received an explanation for the test	94%	16	95%	21	17%	6	50%	4	83%	12	86%	21	22%	0.43	3%	0.83
Proportion of women reporting having had any clinical procedures performed	90%	63	75%	106	75%	136	58%	142	67%	109	82%	103	0.3%	0.99	25%	0.10
Proportion of women reporting consent being sought before procedures were performed	68%	57	68%	79	63%	102	61%	83	81%	73	77%	84	−7%	0.69	2%	0.94
Proportion of women having been encouraged to ask questions	65%	63	50%	105	35%	136	36%	142	53%	109	61%	103	17%	0.33	23%	0.25
Proportion of women having been offered to have a guardian	47%	62	39%	102	21%	136	23%	142	39%	109	33%	103	8%	0.60	−2%	0.92
Proportion of women reporting their privacy & confidentiality being protected	95%	63	96%	106	88%	136	92%	142	88%	109	89%	103	1%	0.94	7%	0.54
Proportion of women reporting blood pressure having been checked	51%	63	39%	106	30%	136	28%	142	52%	109	56%	103	9%	0.73	10%	0.74
Proportion of women reporting baby’s weight was checked	90%	63	74%	106	80%	136	70%	142	69%	109	75%	103	6%	0.64	23%	0.27

DID = effect estimate based on difference-and-difference regression; BL-ML = comparison between cohorts at baseline and mid-term; BL-EL = comparison between cohorts at baseline and end-term; Sig. = significance level of effect estimate

##### Effect of RBF4MNH on perceived quality of care

We observed no statistically significant change attributable to the RBF4MNH on the quality of care ratings for any of the three dimensions measured (technical care, quality of amenities, interpersonal relations) for any of the three sets of services observed (L&D, ANC, PNC) (Table 7). We did, however, observe a small decline in perceived quality of care scores in intervention facilities over time, but in the absence of statistical significance, we cannot exclude the possibility that this decline is attributable to sampling errors.

**Table 7:** Impact of RBF4MNH on perceived quality of care for labour and delivery, ANC and PNC services

Indicator	Baseline				Midline				Endline				DID adjusted BL-ML	Sig.	DID adjusted BL-EL	Sig.
	Control		Intervention		Control		Intervention		Control		Intervention					
	Mean	*N*	Mean	*N*	Mean	*N*	Mean	*N*	Mean	*N*	Mean	*N*				
Labour and delivery services																
Mean score of women’s perceptions on interpersonal relations	9.3	52	9.2	68	9.3	89	8.7	120	9.3	62	8.9	85	−0.5	0.13	−0.1	0.70
Mean score of women’s perceptions on quality of amenities	9.3	51	9.5	68	9.5	89	9.2	120	9.3	62	9.2	85	−0.6	0.07	−0.3	0.45
Mean score of women’s perceptions on technical care	9.3	51	9.2	68	9.3	89	8.8	120	9.1	62	8.7	85	−0.5	0.22	−0.1	0.85
Antenatal care services																
Mean score of women’s perceptions on interpersonal relations	9.2	152	9.1	127	9.2	223	8.9	244	9.2	133	9.3	135	−0.2	0.51	−0.2	0.56
Mean score of women’s perceptions on quality of amenities	9.5	152	9.4	127	9.4	223	9.2	244	9.4	133	9.2	135	−0.1	0.71	−0.2	0.54
Mean score of women’s perceptions on technical care	9.2	152	9.0	127	9.1	223	8.8	244	9.0	133	8.7	135	−0.1	0.74	−0.2	0.39
Postnatal care services																
Mean score of women’s perceptions on interpersonal relations	9.1	63	9.0	105	9.0	136	8.9	142	9.2	109	9.0	103	−0.1	0.73	−0.30	0.45
Mean score of women’s perceptions on quality of amenities	9.3	63	9.2	105	9.3	136	9.2	142	9.3	109	9.1	103	−0.1	0.76	−0.49	0.14
Mean score of women’s perceptions on technical care	8.9	63	8.8	105	9.0	136	8.8	142	8.7	109	8.6	103	−0.1	0.78	−0.31	0.38

DID = effect estimate based on difference-and-difference regression; BL-ML = comparison between cohorts at baseline and mid-term; BL-EL = comparison between cohorts at baseline and end-term; Sig. = significance level of effect estimate

### Qualitative findings

#### Respondents’ characteristic

Women’s age for both IDIs and FGDs varied between 15 and 43 years. The majority of the women were married, literate, had between 1 and 3 children, and all had experienced care at the sampled facilities. The majority of the providers interviewed had worked at the sampled facility 2 to 6 years. All except one had a midwifery diploma.

Appraising the quantitative findings in relation to the qualitative findings both at mid and end terms suggests that women’s perceptions of quality of care on the three aspects (i.e. technical care, quality of amenities and interpersonal relations) varied. In contrast with the unanimously positive judgement that emerged from the quantitative scores, the qualitative analysis revealed women’s heterogeneous view on quality with some aspects being appreciated and others still being heavily criticized. Over the two data collection rounds, we observed an increased appreciation for services delivered at RBF4MNH facilities as well as a capacity to attribute the changes experienced directly to the intervention.

### Perceptions of quality changes in technical care (clinical care)

Although the quantitative findings did not show any measurable change on women’s perception of technical care (Table 7), women in our qualitative sample reported that over time they experienced improvements in technical aspects of service delivery when accessing care at RBF4MNH facilities. Most women who utilized services from RBF facilities perceived providers to be competent to carry out different technical care activities.

*“Things have changed, like when I was delivering this baby, the nurse was there to assist me. I went to the facility late at night. But before taking off my clothes the baby already came out and had suffocated. But the nurse helped me a lot and the baby got better … I never thought the baby would survive, but they assured me of my baby’s survival”.* (Woman in FGD, RBF4MNH health facility, end-term)

Furthermore, when comparing women’s narratives of the provider patient encounter between midterm and end-term data collection rounds at intervention facilities, we noticed a substantial increase in the proportion of women who reported to have been clinically examined, received medication, and received explanations for the medications.

*“They do not explain everything most of times we are told to lay on the bed and they examine what they know without informing us. And after examination we are given medication”.* (Woman in FGD, RBF4MNH health facility, mid-term)

*“After ANC Examination, the health worker informed me well about the condition of the baby and me. The health worker also asked me if the baby was kicking in the womb and I said yes. And thereafter I was given information about the medication and they taught me the importance of the medication. They said that it helps to increase blood because during delivery women lose blood. So I was encouraged to take medication as it was prescribed to me. In fact, the provider was a caring person. But I do not know if my fellow women were also treated like this”.* (Woman in FGD, RBF4MNH health facility, end-term)

Providers at intervention facilities confirmed women’s observations by indicating that they felt more secure in their skills and by reporting greater adherence to recommended standards of care following the introduction of the RBF4MNH Initiative. The vast majority of providers at intervention facilities attributed the change to the trainings and increased supervision offered under the RBF4MNH Initiative.

*“… most of the training which I have done it’s because of RBF, neonatal and maternal care, is been done because of RBF, as of now, am competent compared to the way I was before. I can manage some of the conditions which were difficult to be managed… I am competent enough to do such things”.* (Skilled birth attendant at an RBF4MNH health facility, end-term)

If on one side, women at intervention facilities reported being examined and treated more accurately, on the other side, women also reported concerns in relation to procedures whose objective was not clear to them. For instance, women repeatedly referred to the more accurate removal of the retained products of conception (an infection prevention procedure) promoted by the RBF4MNH Initiative as “mopping the uterus” and at times attributed a sinister meaning to it.

*“In the previous deliveries, they were not cleaning our womb and the remaining things were coming out without them cleaning us. They were only giving us injection after delivery. So I complained to them that the process is painful and the health worker responded to me that this is going to help me to have good health when I get home”.* (Woman in FGD, RBF4MNH health facility, end-term)

Similarly, women displayed little appreciation for some procedures, such as consent seeking, which increased across intervention and control facilities over time (Tables 4, 5 and 6). When asked consent seeking, women postulated concerns that this was necessary at all, since to them, seeking care was per se an expression of consent.

*“When one is going for ANC you are prepared knowing that where I am going I will be touched in such a way. So I feel there is no need for consent seeking”.* (Woman in FGD, RBF4MNH health facility, end-term)

While women at intervention facilities reported considerable improvements in ANC and delivery care, women at both intervention and control facilities continued to be dissatisfied with PNC. Most women indicated that at the PNC encounters, they received no care and even babies were checked only superficially.

*“I did not spend much time during the PNC consultation because the health worker only checked the navel of my baby”.* (Woman in FGD, non-RBF4MNH health facility, mid-term)

*“When I went to the hospital to show my child after one week, the nurse just looked at my child and told me that my child was fine. The child was never weighed and the navel was never checked to see if it had healed”.* (Woman in FGD, RBF4MNH health facility, end-term)

*“When I went to the hospital to show my child after one week, the nurse just looked at my child and told me that my child was fine. The child was never weighed and the navel was never checked to see if it had healed”.* (Woman in FGD, RBF4MNH health facility, end-term)

### Perceptions of quality changes in amenities (service infrastructure)

While respondents from control health facilities complained of scarcity of basic resources, respondents from intervention facilities appreciated the availability of equipment, drugs, enhanced visual privacy (through the use of screens) and infection prevention supplies at both mid-term and end-term periods.

“*The equipments are not adequate. We are told to bring a razor blade, plastic sheet/paper and a piece of cloth. There is also need to add the number of nurses at the facility”.* (Woman in FGD, non-RBF4MNH health facility, mid-term)

*“Our facility is different from how it used to be. Now the environment is looking good. Resources and equipment are also available. Previously, when some of us delivered there, we were told that the facility lacked important equipment.”* (Woman in FGD, RBF4MNH health facility, end-term)

Women at intervention facilities also appreciated positive changes in hygiene and cleanliness, which were not reported by women at control facilities.

*“Compared to the past, there is cleanliness in the facility. It is well mopped and clean. There is no bad smell like in other hospitals. The cleaners are really doing a good job. They clean the bathrooms and the toilets in the morning and in the evening leaving the premises clean as they knock off [..]. There are even bins inside which are used to throw in any trash and this is also enhancing the cleanliness at the hospital”.* (Woman in FGD, RBF4MNH health facility, end-term)

Most providers agreed with the women’s observations and indicated that an increased availability of resources (i.e. drugs and supplies) and infrastructure upgrades due to the RBF4MNH Initiative had made it possible for them to improve service delivery. Despite the reported improvements, most providers at BEmOC intervention facilities indicated that they still struggled for bed space due a high caseload and to the increase in demand which followed the introduction of the RBF4MNH.

*“[…] we have three beds, but we actually have most of the times seven, six, five women delivering at one time – three on the bed, two or three or four on the floor. So when helping a woman while on the floor, sometimes we take risks and it’s so tiresome. That’s the challenge.”* (Skilled birth attendant at an RBF4MNH health facility, end-term)

### Perception of changes in interpersonal care (provider-patient interactions)

A comparison between mid-term and end-term qualitative data provides an indication of improvements in provider-patient interactions in RBF4MNH facilities. At mid-term, the majority of women both at intervention and at control facilities recounted being verbally abused during childbirth.

*“… the nurse pushed me out of the door and asked me the number of deliveries I have ever had and I informed her that this was my sixth delivery. She shouted at me, said that I was lying, “this is your tenth delivery and with the problem you have* [the woman was HIV positive] *you are not supposed to give birth again”. And I was told to deliver alone. Fortunately I delivered a live baby. Then the nurse came and took care of the baby. So this was painful to me”.* (Woman in FGD, RBF4MNH health facility, mid-term)

*“The health workers are mistreating us. For instance when a patient has called for help they ignore by saying I was not there when you were having sex with your husband”.* (Woman in FGD, non-RBF4MNH health facility, mid-term)

At end-term, most women from intervention facilities reported positive experiences in relation to their interaction with providers. However, some women reported continued instances of disrespect and verbal abuse by some selected providers whereby unpalatable language was used.

*“… they were busy passing us and shouting, using obscene language telling me to dress up, saying, “go away, don’t show us your dirty and stinking…” they speak such kind of languages; we have no choice but to endure such abuse, since there is nothing we can do… when we are in the labour room, what we need is just to be assisted. So we put up with their actions, because we know we can’t talk back since they are doctors, we fear that if we answer back they may never help us …”* (Woman in FGD, RBF4MNH health facility, end-term)

Further, both at mid-term and at end-term and both in intervention and control facilities, a few women reported having been ignored or not monitored during labour, ending up giving birth alone or with assistance from cleaners or guardians. Many women criticized a common tendency among providers to send them back from labour wards towards the waiting premises without first examining them. Therefore, some women reported to have given birth outside the labour ward or having been rushed back into the labour ward, and in some instances, babies were reported to have fallen on the floor.

*“… but when she was getting back on her bed, just after her one leg reached the bed, the baby came out with force and fell on the floor, it was me who screamed to the provider saying the girl has delivered and the baby has fallen down. To my dismay, instead of the provider rushing to pick the baby, the provider stood and started shouting at the girl. “Didn’t you know that down there something is coming out” the baby was there down rolling. It was me who told the girl, “Girl, if you have some energy please get down and carry your baby, it’s your baby”. The girl got down and picked up her baby…*.” (Woman in FGD, RBF4MNH health facility, end-term)

In response to instances of verbal abuse, providers confirmed that indeed there are a few “rotten apples” that tarnish the image of all health providers. However, providers at intervention facilities indicated that through the client satisfaction surveys introduced by the RBF4MNH Initiative, facilities receive feedback on issues of concern (e.g. verbal abuse) and make necessary changes to incorporate women’s voices in health care provision.

*“yaa..For us staff we usually not speak well so because of that the mothers keep this in their mind that when I will go to the hospital this is the treatment I am going to get”.* (Skilled birth attendant at an RBF4MNH health facility, mid-term)

*“I can say may be we have improved a little because when women get discharged they respond to a questionnaire asking them what challenges they have met and the experience they had in the past pregnancy and now. Now care is improved a little more compared to the past pregnancy even though there are some hiccups they say this”.* (Skilled birth attendant at an RBF4MNH health facility, end-term)

In response to neglected care, many providers in both intervention and comparison facilities indicated that monitoring the women was difficult due to large workloads, especially because they were running integrated care services: maternal care (i.e. labour and delivery, ANC and PNC), family planning clinics and blood screening (e.g. for HIV and syphilis). They pointed out that in cases where a provider is working alone, they failed to give exclusive attention to labouring women since they were taking care of other services. As a result, some women could not be closely monitored and sometimes, though not often, they would give birth unattended. Providers themselves recognized this as a major hindrance to ensuring quality in service delivery, especially at CEmOC facilities where workload is greater.

## Discussion

The fundamental question addressed in this study was whether the RBF4MNH Initiative (including both PBF and CCT) had an effect on women’s experiences and perceptions of quality of maternal and newborn care services. In the context of this study, we explored three dimensions of women’s perceived QoC: technical care (clinical care); quality of amenities in the facilities, and quality of interpersonal relations (provider-patient interactions) and measured each dimension along the continuum of care from ANC to L&D to PNC. In addition, we reconstructed women’s experiences of care through a detailed structured recall of women’s interaction with providers. We complemented both sets of quantitative data with qualitative data originating from interviews and FGD with women and their providers.

Our quantitative analysis did not detect a significant effect of the RBF4MNH on any of the three dimensions of quality or for any of the services observed. Similarly, our models only detected a handful of significant effects on women’s experiences of care. These findings appear surprising in the light of the fact that qualitative findings are indicative of a much more complex scenario, characterized by a mixture of positive and negative experiences and perceptions in relation to the quality of service provision and to the changes produced by the RBF4MNH Initiative. In spite of reporting considerable improvements following the introduction of the RBF4MNH Initiative, women noted that instances of disrespect, at times leading to overt abuse, continued to overshadow their experiences of care.

This apparent incongruence between quantitative and qualitative findings deserves attention and requires that we take a critical look at the tools we used to quantify experiences and perceived quality of service provision, drawing some important lessons for future research. The lack of effect observed across all scores measuring quality of care can largely be attributed to the high values already recorded at baseline. Detecting further improvements with an average baseline value of 9 would have required either a much larger sample (allowing us to detect changes of even small magnitude as significant) or a change of a considerable magnitude (with women consistently rating quality of care at 10). In addition, as noted in a prior publication [43] and in line with what was observed elsewhere [51], these consistently high ratings are an indication that rural Malawian women struggled to rate quality of service provision along a quantitative scale. In spite of our explicit efforts to develop a culturally sensitive tool (including the use of a visual aid to facilitate rating) [43], this difficulty calls into question the applicability of this approach in rural SSA and should encourage researchers to develop alternative quantitative tools to elicit ratings in these settings beyond the ones currently available.

An additional methodological considerations to be taken into account when appraising our quantitative findings derives from the fact that we cannot exclude that women’s high ratings of quality of service provision might have been influenced by fear of future repercussions from service providers, since we conducted our exit interviews at the health facilities [43]. Furthermore, we acknowledge the potential of a Hawthorne effect [52], with providers modifying their behaviour for the better during our limited stay at the facility. We also acknowledge that women might have faced difficulties in properly recounting and assessing some dimensions included in our structured survey (e.g. health workers honesty, competency, co-ordination and sufficiency of equipment in the delivery room), since during a structured interview, the enumerator does not engage extensively with respondents to contextualize questions and probe for answers, as it is instead the case during a qualitative encounter [41].

The inability to detect an effect on women’s experience of care may also be influenced by our study design and by the overall socio-economic context surrounding the implementation of the RBF4MNH Initiative. A closer look at the quantitative findings on women’s experience of care suggests that change occurred on several dimensions, but did so in an equal manner across intervention and control facilities (e.g. probability of having been clinically examined during L&D). Such an observation normally indicates that change was influenced by a secular trend and that a given intervention did not produce any additional benefit. In our specific case, however, we have reason to believe that the secular trend itself was influenced by the intervention, since, as described earlier, the RBF4MNH Initiative included incentives beyond the single facilities, targeting directly the District Health Management Teams. These incentives, linked to the management team’s supervisory role and to the overall district performance, are likely to have produced changes across intervention and control facilities, since the management teams worked to improve overall performance at a district level. Selecting control facilities outside the concerned districts would have allowed us to bypass this challenge and better discern the effect of the intervention, but this strategy was not feasible due to political and operational concerns (other health interventions supported by other development partners were planned in neighboring districts, thus the MoH could not grant us permission to include them as controls). At the same time, one needs to consider that the decline observed on some dimensions of care across intervention and control facilities (e.g. probability of receiving medication during PNC) was probably due to the fact that the RBF4MNH Initiative was implemented at a time when Malawi was suffering from a major economic crisis [53] during which public resources for the health sector shrank considerably, further limiting supplies for intervention and control facilities alike. These considerations are not meant to cast doubt on the validity of our study, but rather to allow the reader to contextualize the quantitative findings, looking for meaning beyond the mere percentage change and statistical significance detected by our models.

We did, however, identify change attributable to the intervention on a few selected items related to women’s experience of care. Apart from the clinical relevance of the single procedures (which is a subject beyond the scope of our investigation in this study), we observed that while the probability of having one’s blood pressure taken during ANC decreased as a function of the intervention, the probability of being asked whether the patient would like to keep a companion during ANC and of receiving drugs during delivery increased. Complementing the quantitative findings, women’s narrative accounts in our qualitative findings provided indications that the greatest improvements in experiences and quality of service delivery were in relation to improved perceived providers’ competence and service infrastructure. In relation to providers’ competence, women appreciated that physical examinations were conducted; procedures and purpose of medication were explained. For service infrastructure, women appreciated that the facilities were kept clean and hygienic and that drugs, equipment and other essential supplies were readily available and that the women’s privacy was respected. These results indicate the potential of the RBF4MNH to improve women’s satisfaction and in turn service use, given that several prior studies have demonstrated perceived staff competency (i.e. knowledgeable and qualified providers) and service infrastructure (i.e. a health facility that is tidy, clean and with adequate drugs and supplies) to be important factors in shaping clients’ satisfaction and service use [29, 54–59].

Providers confirmed women’s reports by explaining how the RBF4MNH Initiative had contributed towards producing these changes. They indicated that the improved availability of resources represented a positive change, especially in a country like Malawi, where equipment and supply shortages represent the primary source of public outcry and healthcare providers’ frustration [35]. Two parallel evaluations, one looking at the impact of RBF4MNH on clinical processes of care and one exploring health-care workers’ motivation in the context of RBF4MNH, confirm that the availability of equipment and supplies eased the pressure on healthcare providers and enhanced their ability to provide quality services (Brenner et al., under review Bull World Health Organ; Lohmann et al., in preparation). Our findings are not surprising and perfectly aligned with the literature indicating that equipped facilities strengthen the health system [60] and improve women’s perceptions regarding maternal care ([69], [61, 62]) and demand for services [59].

In spite of the improvements experienced as a consequence of the RBF4MNH Initiative, it is worrisome and worth the consideration of policy makers that women continued to report instances of disrespect and abuse (D&A), with some women reporting being verbally abused and neglected even under RBF4MNH. Beyond the need to provide care that meets high clinical standards, the international maternal care community unanimously recognizes women’s right to be treated with respect throughout the delivery and childbirth experience [63]. Consensus on the value of providing respectful care has emerged as the literature confirmed that the display of D&A behaviours by providers may instil fear in women [60] and affect their satisfaction and subsequent use of the health services [60, 64, 65]. Further, neglectful behaviour may instil a feeling of hopelessness which may lead to postpartum depression or stress disorder [66, 67].

The health-care providers’ interviews draw a differentiation between neglectful and abusive behaviours. On the one side, they recognized that perceived neglect may arise as they are called to assist to too many patients at once, thus they simply lack capacity to monitor each labouring woman closely. They explained that the human resource investments brought about by the RBF4MNH were not sufficient to compensate for the increase in workload that followed the implementation of the intervention. This concern with workload needs to be addressed in a prompt manner for the good of clients, but also for the good of providers, since the literature already indicates how providers’ frustration with inefficiencies in health system structures can easily lead to poor patient-provider interactions [60]. On the other side, the providers we interviewed distanced themselves from the instances of abuse reported by women and attributed them to single providers, who are accused of “spoiling” the name of all providers in the country.

Given the absence of comparable studies exploring experiences of care and perceptions of quality of service delivery within the context of a PBF intervention, it is difficult to gauge to what extent the concerns raised by women in our study are shared in other settings where PBF has been implemented. To our knowledge, however, currently active PBF programs, including the RBF4MNH Initiative, reward improvements in infrastructural and process dimensions of care, but still struggle to set incentives to target directly the provider-patient interaction. As such, in the absence of a specific strategy targeting the interpersonal dimension of care, PBF is unlikely to motivate a behavioural change that leads to improvements in the provider-patient interactions. This difficulty is further enhanced by the fact that providers engaged in PBF programs often face an increased demand for services. In a setting with relatively high baseline workload such as Malawi, resulting excessive workload can thus pose a threat to the motivational benefits generated by the incentives, the increased autonomy and the supervision granted by PBF, as for instance reported from Rwanda [68] and Nigeria [69].

The fact that our results on interpersonal relations reflect the status quo (that of D&A) as observed in the literature [37, 38, 61] suggests that D&A is a common concern in Malawi and that the RBF4MNH did not include a sufficient number of instruments to counteract this phenomenon. This is somewhat surprising given the emphasis placed by the programme on hearing women’s voices, for instance through the inclusion of client exit interviews (satisfaction surveys) in the programme’s regular review cycle. Therefore, we urge policy makers and project implementers to consider additional means/specific indicators to counter D&A and stimulate provision of respectful maternal care. Discussion should be initiated in this RBF as well as in parallel ones across SSA on how to integrate an additional set of targets and related indicators to promote provision of respectful maternal care.

## Conclusion

Overall, we found no effect of the RBF4MNH Initiative on perceived quality of care. However, we did find intervention effects on certain elements of women’s experience of care. Our qualitative results also suggest positive improvements in respect to availability of equipment, drugs, supplies, and facility cleanliness. However, there were continued instances of D&A even under the RBF4MNH Initiative, largely influenced by perceived staff shortages and high workload on the part of the providers. Current and future RBF interventions should ensure that besides the provision of material resources, the quantity of human resources available to provide quality care should be sufficient. In addition, issues of D&A deserve attention from policy makers. RBF interventions should consider incentivising positive attitudes among providers and adopt rights-based-approaches to defend human dignity and provide women with their basic fundamental human rights [55].

## Abbreviations

ANC: Antenatal Clinic
CCT: Conditional Cash Transfer
CEmOC: Comprehensive Emergency Obstetric Care
COMREC: College of Medicine Research and Ethics Committee
DCs: District Commissioners
DHOs: District Health Officers
DID: Difference in Difference
EmONC: Emergency obstetric and neonatal care
FGD: Focus Group Discussion
IDI: In-Depth Interviews
IP: Infection prevention
LMICs: Low and middle income countries
MMR: Maternal Mortality Ratio
MNH: Maternal and Newborn Health
MoH: Ministry of Health
NMR: Neonatal Mortality Rate
OLS: Ordinary Least Squares
PBF: Performance Based Financing
PNC: Postnatal care
QoC: Quality of care
RBF4MNH: Results Based Financing for Maternal and Newborn Health
RHU: Reproductive Health Unit
SES: Socio Economic Status
SSA: Sub Saharan Africa
